# Oesophageal cancer among the Turkomans of northeast Iran

**DOI:** 10.1054/bjoc.2000.1414

**Published:** 2000-11

**Authors:** F Saidi, A Sepehr, S Fahimi, M J Farahvash, P Salehian, A Esmailzadeh, M Keshoofy, N Pirmoazen, M Yazdanbod, M K Roshan

**Affiliations:** Department of Surgery, Modarress Hospital, Beheshti University School of Medicine, Tehran, Iran; Massieh Daneshvari Hospital, Tehran, Iran; Department of Gastroenterology, Tehran University School of Medicine, Tehran; Department of Pathology, Iran University School of Medicine, Tehran; Department of Surgery, Free Islamic University, Tehran; Massieh Daneshvari Hospital, Tehran; Department of Surgery, Tehran University School of Medicine, Tehran, Iran; International Agency for Research on Cancer, Lyon, France

**Keywords:** oesophageal cancer, oesophageal balloon cytology, active surveillance, Turkoman Plain

## Abstract

A Caspian Littoral Cancer Registry survey in the early 1970s established northern Iran as one of the highest oesophageal cancer incidence regions of the world. To verify this, an oesophageal cancer survey was carried out between 1995 and 1997 in the Turkoman Plain at the southeastern corner of the Caspian Sea. Oesophageal balloon cytology screening was carried out on 4192 asymptomatic adults above age 30 years in one town and three adjoining villages with a total population of 20 392 people at risk. Oesophagoscopy was performed on 183 patients with abnormal cytological findings. The discovery of two asymptomatic small squamous cell cancers and one ‘carcinoma- suspect’ implied a prevalence ranging from 47.7 per 100 000 to 71.5 per 100 000. During a 1-year active surveillance, 14 patients were found with clinically advanced oesophageal squamous cell cancer, yielding age-standardized incidence rates of 144.09 per 100 000 for men and 48.82 per 100 000 for women. The very high frequency of oesophageal cancer reported for northern Iran 25 years ago stands confirmed. Differences in incidence rates, then and now, can be attributed to survey methods used and diagnostic criteria applied, but not to socioeconomic factors, which have remained relatively stable. Oesophageal balloon cytology is a practical method of mass screening for oesophageal cancer in Iran. © 2000 Cancer Research Campaign


*British Journal of Cancer* (2000) **83**(9), 1249–1254
© 2000 Cancer Research Campaign
doi: 10.1054/ bjoc.2000.1414, available online at http://www.idealibrary.com on IDEAL®

### Oesophageal cancer among the Turkomans of northeast Iran

**F Saidi[1], A Sepehr[2], S Fahimi[1], MJ Farahvash[3], P Salehian[4], A Esmailzadeh[5], M Keshoofy[6], N Pirmoazen[1], M Yazdanbod[7] and MK Roshan[3]**

[1]Department of Surgery, Modarress Hospital, Beheshti University School of Medicine, Tehran, Iran; [2]Massieh Daneshvari Hospital, Tehran, Iran; International Agency for Research on Cancer, Lyon, France; [3]Department of Gastroenterology, Tehran University School of Medicine, Tehran; [4]Department of Pathology, Iran University School of Medicine, Tehran; [5]Department of Surgery, Free Islamic University, Tehran; [6]Massieh Daneshvari Hospital, Tehran; [7]Department of Surgery, Tehran University School of Medicine, Tehran, Iran

**Summary** A Caspian Littoral Cancer Registry survey in the early 1970s established northern Iran as one of the highest oesophageal cancer incidence regions of the world. To verify this, an oesophageal cancer survey was carried out between 1995 and 1997 in the Turkoman Plain at the southeastern corner of the Caspian Sea. Oesophageal balloon cytology screening was carried out on 4192 asymptomatic adults above age 30 years in one town and three adjoining villages with a total population of 20 392 people at risk. Oesophagoscopy was performed on 183 patients with abnormal cytological findings. The discovery of two asymptomatic small squamous cell cancers and one 'carcinoma- suspect' implied a prevalence ranging from 47.7 per 100 000 to 71.5 per 100 000. During a 1-year active surveillance, 14 patients were found with clinically advanced oesophageal squamous cell cancer, yielding age-standardized incidence rates of 144.09 per 100 000 for men and 48.82 per 100 000 for women. The very high frequency of oesophageal cancer reported for northern Iran 25 years ago stands confirmed. Differences in incidence rates, then and now, can be attributed to survey methods used and diagnostic criteria applied, but not to socioeconomic factors, which have remained relatively stable. Oesophageal balloon cytology is a practical method of mass screening for oesophageal cancer in Iran. © 2000 Cancer Research Campaign

**Keywords**: oesophageal cancer; oesophageal balloon cytology; active surveillance; Turkoman Plain

Northeastern Iran was recognized as a very high incidence area for oesophageal cancer almost 30 years ago, based on the results of a large-scale cancer survey carried out in the Caspian Littoral in the early 1970s (Kmet and Mahboubi, 1972; Mahboubi et al, 1973). An exceptionally high incidence of cancer of the oesophagus was found for both men and women in the relatively dry regions east of the Caspian Sea, and a much lower incidence in the more humid western parts of the Caspian Littoral.

Two early-detection studies also showed a high prevalence of pre-cancerous lesions in the areas of highest incidence (Dowlatshahi et al, 1978; Crespi et al, 1979). Other studies identified the role of suspected aetiological factors (Pour and Ghadirian, 1974; Hormozdiari et al, 1975; IARC, 1975; Kirk et al, 1976; Joint Iran–IARC Study Group, 1977; Hewer et al, 1978; Mahboubi and Aramesh, 1980; Cook-Mozaffari et al, 1979; Farhud et al, 1979; Hashemi et al, 1979; O'Neill et al 1980; Kmet et al, 1981) showing the highest risk to be among the traditionally semi-nomadic Turkoman population. There also seemed to be an association with a very restricted diet from infancy (low in animal protein and fruit or vegetables), consumption of dry, coarse bread contaminated with sharp seeds and silica fibres and of hot tea, and the regular smoking or swallowing of opium dross ('sukhtheh') which was shown to be mutagenic (Hewer et al, 1978; Dowlatshahi and Miller, 1985). Cigarette smoking and alcohol consumption, major risk factors among heavy drinkers and smokers in western populations (Munoz and Day, 1996), was on a negligible scale among men in the high-incidence areas, and practically absent among women. Intensive investigation of foodstuffs for known carcinogens such as nitrosamines, aflatoxins or polycyclic hydrocarbons showed very low occurrence and no association with patterns of oesophageal cancer incidence in this region.

*Received 27 March 2000*
*Revised 19 June 2000*
*Accepted 27 June 2000*

*Correspondence to:* F Saidi

Other epidemiological studies and investigations on the role of 'sukhteh' (Ghadirian et al, 1985), as well as intervention studies using vitamin supplements to see if a reduction in pre-cancerous lesions could be achieved, were in the offing. They came to a halt, however, with the sociopolitical events occurring in Iran from 1979 onwards.

The present study by the Iranian Society for the Study of Esophageal Cancer (ISSEC) set out to re-evaluate the incidence of oesophageal cancer among the Turkomans of northeast Iran two and a half decades after the initial survey. An opportunity arose, also, to assess the feasibility of the balloon cytology technique as a mass screening tool for oesophageal cancer in this region.

### MATERIAL AND METHODS

It was beyond the scope of ISSEC to repeat a protracted cancer registry survey covering the general five Turkoman areas defined in previous studies. Instead, a mass-screening programme for adults over the age of 30 years in a localized population was planned, using the balloon cytology method, followed by endoscopic examination of suspected cases. In addition to the screening survey, an active search was made in the same population over a period of 1 year, for newly diagnosed cases of oesophageal cancer. ISSEC members agreed to arrange for free follow-up services and treatment of all patients identified during the survey period. Approval for the study was obtained from the Ethics Committee of the Research Department of the Ministry of Public Health, and informed consent of all participants was obtained for all intrusive procedures.

### Mass screening programme

### *Oesophageal balloon cytology examination*

This programme was undertaken in the Turkoman Plain, an 1860 $km^2$ flat area located at the south-eastern corner of the Caspian Sea, targeting the largest town of Bandar-e-Turkoman, and the three nearby villages of Khaje-nafas, Sijaval and Panj Peykar, with a total population of 65 265 by official 1994 Census, with 20 392 people over the age of 30 years.

Preliminary meetings were held with local officials and religious leaders to secure the tactical cooperation of the former and moral support of the latter. The purposes and methods of the project were announced after Friday mosque prayers, and actual balloon intubation technique demonstrated at local clinics and in two high schools, asking students at the latter to encourage their parents to participate in the screening programme.

This part of the project was undertaken in two phases during the first week of October of two successive years (1995 and 1996), to suit the academic schedule of 70 senior medical students from Tehran, who undertook the practical aspects of the screening programme under the supervision of clinical members of ISSEC. An unusually heavy rainfall throughout the six days of the October 1996 survey resulted in fewer participants being recruited during this second phase of the study.

The screening programme was publicized by radio and television, by posters and by announcement after Friday prayers. All adults aged 30 years and over were invited to attend, in a fasting state, one of eight clinics set up at convenient locations in the town of Bandar-e-Turkoman. Roving cars with loudspeakers announced that transport was available for the elderly. In the October 1996 phase of the screening, the only way to examine new volunteers was for the survey team to wade to their homes.

Each of the eight clinics was manned by six to eight medical students who obtained informed consent from arriving volunteers, administered a questionnaire/clinic form covering general health, ethnic affiliation, family history of oesophageal cancer and dietary habits, and performed physical examinations. The students also performed actual intubation under supervision of clinical members of ISSEC, using disposable samplers, and prepared slides from specimens. Arriving volunteers who gave a history of progressive dysphagia were not intubated but were referred to the local 90-bed general hospital for barium swallow radiography. Also excluded were any persons found, during physical examination, to have respiratory or cardiac insufficiency or other serious medical conditions.

Chinese-type oesophageal balloon cytology tubes (Roth et al, 1997) were tested in a pilot investigation in the town of Gomishan and the neighbouring village of Banavar further to the north, in the Turkoman Plain. A total of 331 adults were intubated on this occasion. While the cellular yield of these samplers was satisfactory, about 10% of participants experienced nausea, vomiting and bloody expectoration. This led to a modified sampler being designed and manufactured by SUPA Co Ltd (Tehran, Iran) incorporating a somewhat smaller latex rubber balloon attached to the tip of a 72 cm single-lumen plastic tube. The tube was marked at 10 cm intervals and its rigidity adjusted to provide enough flexibility for controlled intubation. The balloon portion, covered with a finely woven thin-strand cotton mesh, measured 0.8 cm × 5 cm in the collapsed state, expanding to a sphere 3 cm in diameter when inflated with 20 cc of air. The new sampler was tested with the help of 10 patients with known cancer of the oesophagus and 20 healthy volunteers, in Tehran. Accurate diagnosis was achieved for all individuals. At first, xylocaine spray was used to facilitate intubation in the main study, but subsequently participants were found to accept the new sampler readily and without topical anaesthesia.

Three glass slides engraved with the participant's identifying number were prepared from each intubation and were then promptly fixed in 99% alcohol for at least 3 min, and sent to Tehran where they were stained by the Papanicoloau technique and examined by trained cytolo-technicians under the supervision of ISSEC cytopathologist. All abnormal slides were re-examined by a second cytopathologist. The Bethesda System (1993) was used to classify and grade the oesophageal mucosal cells.

### *Endoscopic examination*

Participants whose oesophageal cytological examination had been read as abnormal squamous cell of undetermined significance (ASCUS) or worse, on the concurrence of two cytopathologists examining the slides independently, were contacted in person and invited to attend, in the fasting state, the local hospital for endoscopic examination. This was performed by members of the ISSEC team. All patients were given topical anaesthetic before introduction of the fibreoptic endoscope, and a 3% Lugol's iodine solution was sprayed into the oesophagoscope through a catheter to enhance visibility. The entire mucosal lining of the oesophagus and stomach was inspected and all gross abnormalities or suspicious areas biopsied. In addition, biopsies were taken from the posterior aspect of the oesophageal mucosa at 2 cm proximal to the gastroesophageal junction and at 38, 30 and 20 cm from the incisors. Specimens were fixed in 10% formalin solution, labelled and taken to Tehran, where slides were prepared and stained with haemotoxylin and eosin.

The slides were each read by two cytopathologists in Tehran, without knowledge of the results of the cytological examination. Nuclear atypia (enlargement, pleomorphism and hyperchromasia), abnormal cell polarity and abnormal tissue maturation were considered indicative of dysplastic changes. Mild dysplasia was diagnosed when such changes were limited to the lower third of the epithelial layer, moderate dysplasia when two thirds, and severe dysplasia when the entire epithelial layer was involved, regardless of its thickness (Dawsey et al, 1994). The diagnosis of squamous cell cancer was based upon the presence of clearly neoplastic cellular changes, as well as actual penetration of the basement membrane. All discrepant diagnoses were resolved by joint review.

### Active surveillance programme

During the year following the second phase of the screening programme in October 1996, a search was made for patients from

*© 2000 Cancer Research Campaign*

**Table 1** Selected patient characteristics of participants in the balloon cytology screening programme

| Characteristic | Percentage |
|---|---|
| Age in years (mean (range)) (SD = 11.9) | 44.1 (30–88) |
| Sex | |
| Male | 47.2 |
| Female | 52.8 |
| Ethnic origin | |
| Turkoman | 65.7 |
| Zaboli-Sistani | 1.5 |
| Mazandarani | 11.5 |
| Other[a] | 21.4 |
| Residence | |
| Town | 78.2 |
| Village | 21.8 |
| Married status | 94.7 |
| Family history of oesophageal cancer | |
| Father | 6.2 |
| Mother | 3.0 |
| Symptoms and signs | |
| Heartburn | 37.5 |
| Anaemia | 17.0 |
| Oral hygiene | |
| Good | 21.5 |
| Fair | 25.5 |
| Poor | 18.9 |
| Bad | 7.5 |
| Dental prosthesis | 26.6 |
| Habits | |
| > 10 cigarettes/day for 5 years | 7.2 |
| Alcohol[b] | 6.9 |
| Opium, in any form[b] | 8.5 |
| Nass[b,c] | 5.4 |
| Dietary history | |
| Salted or smoked fish frequently | 59.9 |
| Fresh fruit | |
| Once in 3 months | 3.1 |
| Once a month | 8.6 |
| Weekly | 88.3 |
| Fresh vegetables | |
| Once in 3 months | 7.4 |
| Once a month | 17.5 |
| Weekly | 75.1 |
| Pickled vegetables occasionally | 75.3 |
| Pica | 6.1 |
| Wheat bread | 99.1 |
| Stale bread | 6.5 |
| Water source | |
| Piped | 96.0 |
| Well | 4.0 |
| Refrigerator > 20 years | 79.7 |

[a] Not considered by local residents as true Turkoman, arrivinig here presumably from Kazakhestan more than 150 years ago; [b] Probably an underestimation because of legal injunctions; [c] Nass is a greenish mixture of 50% tobacco powder, 20–35% filtered burnt wood-ash, 10–20% calcium hydroxide and 5–10% arsenic oxide the study population who had overt oesophageal cancer. Local health officials were contacted for information on any patient suspected of being afflicted, and the director of the local hospital, himself a Turkoman, painstakingly contacted families in search of such patients who had sought medical care outside the Turkoman area. X-ray files in the local hospital and medical records at community hospitals in Gorgan and Gonbad, the two largest towns adjacent to the Turkoman area, and at three hospitals in Tehran likely to have admitted Turkoman patients, were reviewed. The close rapport established with the local population in the course of the screening programme facilitated this active surveillance programme.

### RESULTS

### Mass screening programme

A total of 4192 participants, or 20.5% of the total study population, were successfully intubated, 3910 in October 1995 and 282 in October 1996. Of these, 1971 were men and 2221 were women, aged 30–88 years. Selected characteristics of the participants are shown in Table 1.

Fifteen potential participants were found to have cardiopulmonary insufficiency and two had cirrhosis of the liver on physical examination and were, therefore, not intubated. Thirty arrivals left after watching other participants being intubated, and 125 were unable to swallow the sampler after three separate attempts, mostly because of heightened gag reflex. The 4192 volunteers who were successfully intubated suffered no greater discomfort than transient mild nausea. No early or late complications ensued. Cellular preparations adequate for cytological diagnosis were available for 99.4% of the study population (4165 individuals). Table 2 shows the distribution of cytological diagnoses, the majority of abnormal ones being squamous cells of undetermined significance (ASCUS). 1.7% showed squamous intraepithelial lesions (0.7% low-grade (LSIL) and 1.0% high-grade (HSIL)). For three subjects, or 0.1% of the total study group, the sampled mucosal cells were indicative of squamous cell carcinoma.

It was intended that all participants with a diagnosis of ASCUS or worse (253 subjects) be asked to undergo an endoscopic examination. In the event, 21 could not be located, 38 were not willing to submit to endoscopy despite repeated encouragement, and 11 subjects were not able to tolerate the procedure because of either discomfort or fear. This left 183 participants for whom a satisfactory examination of the oesophagus and stomach could be performed and from whom biopsy specimens were taken.

The histological diagnoses are given in columns 4–10 of Table 2, cross-tabulated with the cytological diagnoses. Fourteen subjects, or 7.7% of those endoscoped, showed normal oesophageal mucosal lining. Thirty-eight subjects had benign lesions, of whom 26 (or 14.2% of those histologically confirmed) had oesophagitis. Dysplasia was seen in 70% of the participants. This was mostly of the mild type, but nine subjects, or 4.9% of all those endoscoped, were diagnosed as having severe dysplasia, including one for whom greater pleomorphism and loss of polarity throughout the epithelial layer was apparent, suggesting a possible carcinoma. However, the orientation of this particular ‘carcinoma suspect’ specimen was sub-optimal, with no basement membrane visible. The relatives of this participant, a 73-year-old woman, would not agree to repeat endoscopy. Two further subjects were found to have invasive tumours, one a poorly differentiated squamous cell carcinoma 1 cm in diameter in the lower oesophagus (35 cm from the incisors) and the other a well differentiated squamous cell carcinoma 0.5 cm in diameter in the mid-oesophagus (25 cm from the incisors).

The cytological diagnosis of the one ‘carcinoma suspect’ patient was ASCUS, while those of the two small invasive carcinomas were HSIL and cancer, respectively. The other subject with a cytological diagnosis of squamous cell cancer who agreed to undergo

© 2000 Cancer Research Campaign

**Table 2** Correlation between cytological and histological diagnoses

| Cytological diagnosis | Patients (n (%)) | Patients endoscoped | Histological diagnosis: Normal | Benign lesions[a] | Mild dysplasia | Moderate dysplasia | Severe dysplasia | 'Carcinoma suspect' | Squamous cell carcinoma | Positive predictive value (of cytological diagnosis) for histological diagnosis of dysplasia or cancer |
|---|---|---|---|---|---|---|---|---|---|---|
| ASCUS | 106 (2.5) | 71 | 6 | 21 | 26 | 13 | 4 | 1 | 0 | 61.9% |
| ASCUS-R | 30 (0.7) | 18 | 1 | 6 | 9 | 2 | 0 | 0 | 0 | 61.1% |
| ASCUS-N | 43 (1.0) | 36 | 4 | 4 | 19 | 9 | 0 | 0 | 0 | 77.7% |
| LSIL | 29 (0.7) | 23 | 0 | 5 | 13 | 5 | 0 | 0 | 0 | 78.2% |
| HSIL | 42 (1.0) | 33 | 3 | 2 | 16 | 7 | 4 | 0 | 1 | 84.8% |
| SCC | 3 (0.1) | 2 | 0 | 0 | 0 | 1 | 0 | 0 | 1 | 100.0% |
| Normal or reactive cells | 3812 (90.9) | – | – | – | – | – | – | – | – | – |
| Inadequate cellular preparation | 127 (3.0) | – | – | – | – | – | – | – | – | – |
| Total | 4192 | 183 | 14 | 38 | 83 | 37 | 8 | 1 | 2 | 71.5% |

ASCUS = Atypical squamous cells of undetermined significance, -R = Favour reactive, -N = Favour neoplastic; LSIL = Low-grade squamous intraepithelial lesion; HSIL = High-grade squamous intraepithelial lesion; SCC = Squamous cell carcinoma; [a]Including oesophagitis (26 cases, 14.2% of all histological diagnoses)

**Table 3** Prevalence of oesophageal squamous cell carcinoma in the asymptomatic adult population over 30 years of age

| Diagnosis | Patients (*n*) | People screened (*n*) | Prevalence per 100 000 |
|---|---|---|---|
| Invasive squamous cell cancer | 2 | 4192 | 47.7 |
| Invasive squamous cell cancer, incuding one 'carcinoma suspect' | 3 | 4192 | 71.6 |

endoscopy was diagnosed as having moderate dysplasia. A third participant who had a cytological diagnosis of cancer, a woman of 54 years, refused endoscopy, but was alive and well 2 years later.

The predictive value of the cytological diagnoses for the presence of abnormal histology (mild dysplasia or worse) as shown in the last column of Table 2, was just over 60% for ASCUS, around 80% for LSIL and HSIL, and 100% for the two cases of carcinoma.

Table 3 shows the prevalence rates of oesophageal cancer in the screened asymptomatic population of 4192 people over the age of 30 years to be 47.7 per 100 000, rising to 71.6 per 100 000 if the one 'carcinoma suspect' case were included.

### Active surveillance programme

A total of 14 patients, eight men and six women, aged 52–80 years, were first diagnosed with clinically advanced cancer of the oesophagus during the year beginning October 1996. A histological diagnosis of squamous cell carcinoma was available for all 14 patients on the basis of endoscopic biopsy, the surgically resected specimen, or both. This diagnosis was established, in all instances, outside the Turkoman region, for eight patients in the Gorgan and Gonbad hospitals and for six patients in Tehran hospitals.

All 14 patients, except one, were considered by local residents as being true Turkomans. None had participated in the oesophageal cytology mass-screening programme of the previous year, for reasons generally expressed as: 'I did not think it important,' or 'it would have been useless, anyway'. By October of 1998, all but two who had been operated upon in Tehran or Gorgan had died.

Since most patients with oesophageal cancer succumb within 1 year from the time the diagnosis is first established on histological grounds, the age-standardized incidence rates for the 14 patients with clinically advanced oesophageal cancer have been calculated as 144.09 per 100 000 for males and 48.82 per 100 000 for females (Table 4).

### DISCUSSION

The 1968–1971 cancer survey of northern Iran was based on an ad hoc cancer registry centre set up jointly by the Institute of Public Health Research of Tehran University and the International Agency for Research on Cancer of Lyon, France. Trained technicians collected data at 4–6 week intervals on all cancers from the offices of about 500 local physicians and clinics serving the population (about 4 million people) of the Caspian Littoral. An initial account of the relationship between ecological features of the region and the incidence of oesophageal cancer was published in 1972 (Kmet and Mahboubi, 1972). The age-adjusted incidence

 *© 2000 Cancer Research Campaign*

**Table 4** Age-standardized, cumulative and crude incidence rates for squamous cell carcinoma of the oesophagus per 100 000 (October 1996–October 1997)

| Incidence | Males | Females | Total[a] |
|---|---|---|---|
| Age-standardized[b] | 144.09 | 48.82 | 94.42 |
| Cumulative (0–80 years %)[c] | 34.41 | 9.99 | 21.68 |
| Crude | 25.54 | 17.68 | 21.45 |

[a] Male/Female ratio = 48/52; [b] Direct age-standardization to world standard population (Doll and Cook, 1967; dos Santos, 1999); [c] Population divided by 10-year age groups rates for cancer of the oesophagus during the first 3 years of registration (Mahboubi et al, 1973) ranged from a high of 165.5 per 100 000 for men and 195.3 per 100 000 for women in the easternmost part of the survey area, to a low of 13.0 per 100 000 for men and 2.3 per 100 000 for women in the western part of the Caspian Littoral. The age-adjusted oesophageal cancer incidence rates for the northern Gorgan area, east of the Caspian Sea and most approximate to the presently surveyed region, were 83.7 per 100 000 for men and 76.9 per 100 000 for women.

The modified balloon sampler used in the present survey proved acceptable to 96% of participants, and the harvested cells were adequate for cytological evaluation in 97% of those tested, with an overall positive predictive value of cytological diagnosis for histological diagnosis of dysplasia or cancer of 71.5%.

Prevalence rates obtained in the present survey cannot be compared directly with the incidence rates of the previous survey, as these were based on asymptomatic participants. Allowance must also be made for possible over-reading of both cytological and histological specimens, as well as some technical errors in procuring and preparing them. However, regardless of whether or not the single ‘carcinoma suspect’ case is included, the range of prevalence obtained in this study supports the premise that the Turkoman population surveyed remains at very high risk for developing oesophageal squamous cell carcinoma.

The incidence of oesophageal cancer obtained in the current study, whether expressed as age-standardized, crude or cumulative rates, verifies in a more direct manner the findings of the study of 25 years ago. Differences between the previous and the present survey are more likely due to morphologic than biologic causes: the latter study covering a smaller population yielded correspondingly fewer cases and less stable rates. Furthermore, the diagnosis of oesophageal cancer in the 1973 report for the nearby Gorgan area, without specifying cell type, was based 83.5% of the time on radiological, 7.6% on clinical and 8.8% on histological grounds. It was stated at that time that, ‘… a fairly confident diagnosis of cancer of the oesophagus can be made by X-ray or even on clinical symptoms alone’ (Mahboubi et al, 1973), and further, ‘We feel, then, that no serious bias has arisen due to lack of histological confirmation’.

The previous survey may have overestimated the true situation as regards oesophageal cancer in northeast of Iran. The present study reflects, on the other hand, an underestimation of the frequency of oesophageal cancer among the Turkomans. The sensitivity of the balloon sampler detecting on a single examination concurrent biopsy-proven carcinoma varies between 14 and 36% (Dawsey et al, 1997). Less than three quarters of patients found to have abnormal cytological diagnosis in the screening programme came to satisfactory endoscopy. And lastly, access to radiological or clinical records in this study was unavoidably incomplete, with an unknown number of oesophageal cancer patients probably escaping detection.

The cytological results of the current study showed 6.0% of the screened population to have abnormal cells. This is similar to the figure of 7.1% found in a neighbouring area of slightly lower incidence in the late 1970s, among 280 people over the age of 20 who were screened by brush cytology (Dowlatshahi et al, 1978), although it is not clear whether that particular study excluded persons who were already affected by dysphagia.

An endoscopic survey carried out in the area of highest incidence on 430 subjects of both sexes aged 15–70 (Crespi et al, 1979) revealed oesophagitis in 87% of those without upper gastrointestinal symptoms – 52% of the total study population. Cytological specimens were taken but not evaluated, so that direct comparison with the current study is not possible.

Apparent or real differences in oesophageal incidence rates between the previous and present survey cannot be ascribed to interim sociopolitical changes. Most Turkoman households had refrigerators and access to piped drinking water 20 years ago. The current questionnaire shows that food habits now are essentially those described in in the latter part of the 1970s (Joint Iran–IARC, 1977; Cook-Mozaffari et al, 1979). No major industries had opened up and piped gas remains the principal cooking fuel. While this region experienced the same doubling of population as the rest of the country over the past three decades, no population shifts had occurred here.

The majority of adult Turkomans seemed to know the disease well and be aware of its grave prognosis. Yet, as had been noted by previous investigators (Dowlatshahi et al, 1978), many chose not to be screened, endoscoped or treated. Another reason for a compliance rate of 20.5% in the screening programme – ignoring inclement weather during its second phase – may have been novelty of the approach plus unfamiliarity of the local population with the balloon intubation technique and those performing it.

The two studies, 25 years apart and different in scope and survey methods used, confirm northeast Iran’s status as a very high incidence area for oesophageal cancer. This conclusion must rest, however, more on the range than exact rates of incidence obtained. The balloon cytology method for oesophageal cancer mass-screening proved practical and cost-effective. The general impression that oesophageal cancer is a common malignancy in other parts of this country as well – first noted in the 1950s and later reinforced by reports from Tehran (Habibi, 1965) and Shiraz (Haghighi et al, 1971; Sadeghi et al, 1977) – has now created interest among public health officials, and an oesophageal and gastric cancer survey has been approved, targeting the Ardebil region of northwest Iran, from autumn of 2000.

### ACKNOWLEDGEMENTS

Other members of the non-profit, non-governmental, Iranian Society for the Study of Esophageal Cancer (ISSEC) are Manuchehr Daii MD, Mohsen Hojjati MD, Ghafour Khajeh MD, Massieh Madani MD, Mahmoud Mahmoudi PhD, Alireza Mohajerani MD, and Abed-Ali Ziq’ee PhD. This study was supported by Grant No 6947 of the Research Department, Ministry of Public Health, Government of Iran. The Olympus Keymed Company Ltd, UK, is thanked for the short-term loan of a complete Olympus oesophagoscopy unit.

*© 2000 Cancer Research Campaign*

The close and effective collaboration of Dr Taj Mohammad Ilmani and Dr Sadegh Ali Azimi are gratefully acknowledged. Appreciation is also expressed to Dr Hossein Malekafzali for unwavering support and guidance. Dr Sanford M Dawsey of the Cancer Prevention Studies Branch, National Cancer Institute, Bethesda, Maryland is acknowledged for valuable discussions and suggestions. Dr Ali Solgi is thanked for mobilizing and supervising the following medical students: Mansour Abbasifar, Mahdi Ahangar-e-Kani, Zargham Ahmadi, Mohsen Ahooghalandari, Alireza Alaghebandan, Behzad Alaghi, Babak Al-e-Agha, Pouyan Alizadeh, Kamyar Amin, Mohammadreza Amjadi, Alireza Azimzadeh-Moghaddam, Hooman Bakshandeh, Pejman Bakhtiari, Babak Behnam, Heydar Dadkhah, Mohammad Davoudi-eroshan, Hossein Delavar-e-kasmaei, Ali Esmatinejad, Afshin Farahanchi, Ali Faraji, Khosrou Fatehi, Aghajan Garkaz, Mohsen Gerami-fahim, Farzin Ghahghaei, Mohieddin Ghofrani, Mohsen Ghofrani, Behzad Ghorbani, Pejman Haddadi, Mehrnaz Hadian, Mohammadreza Heidari, Alireza Iranpoor, Hamid Jahani, Mohammad Joz-e-abhari, Mohammad Kalantari, Hadijipikhi Kam, Serajeddin Kam, Kambiz Karimi, Mazdak Khalili-e-boroud-jeni, Mahdi Khalili-e-samani, Sina Madani, Amirhossein Mani, Homayoon Mansourpour, Alireza Milanifar, Pejman Mirzakhani, Bahram Moghassem, Kamran Mohammadi-e-Joneidi, Mehrdad Moradi-madani, Farshid Moshiri, Mahdi Najjarian, Arash Naseri, Ali Naseri, Alireza Nooralishahi, Peiman Noshiavanpoor, Farbod Rahnamai-e-Chisaz, Mohammad Rashighi, Payam Razavi, Alireza Rostami, Kiarash Saatchi, Reza Saghebi, Hossein Sakhipoor, Mohsen S. Seidsalehi, Shahrooz Shadroo, Amir Shaebani, Majid Shakiba, Ahmadreza Shamshiri, Soroush Sohrabi, Hossein Sotoodehravan, Omid Soufinia, Hossein Tadjdar and Behzad Vahdani.

### REFERENCES

The Bethesda System (1993) for reporting cervical/vaginal cytologic diagnosis. *Acta Cytologica* **37**: 115–124

Cook-Mozaffari PJ, Azordegan F, Day NE, Ressicaud A, Sabai C and Aramesh B (1979) Esophageal cancer studies in the Caspian Littoral of Iran: results of a case-control study. *Br J Cancer* **39**: 293–308

Crespi M, Grassi A, Amiri G, Munoz N, Aramesh B, Mojtabai A and Casale V (1979) Oesophageal lesions in northern Iran: a premalignant condition? *Lancet* **2**: 217–221

Dawsey SM, Lewin KI, Fu-Sheng L, Wang GQ and Shen Q (1994) Easophageal morphology from Linxian, China. Squamous histologic findings in 754 patients. *Cancer* **73**: 2027–2037

Dawsey SM, Shen Q, Nieberg RK, Liu SF, English SA, Cao J, Zhou B, Wang GQ, Lewin KJ, Liu FS, Roth MJ and Taylor PR (1997) Studies of esophageal balloon cytology in Linxian, China. *Cancer Epidemiol Biomarkers Prev* **6**: 121–130

Doll R and Cook P (1967) Summarizing indices for comparisons of cancer incidence data. *Int J Cancer* **2**: 269–279

Dos Santos I (1999) *Cancer Epidemiology: Principles and Methods*. International Agency for Research on Cancer: Lyon

Dowlatshahi K, Daneshbod A and Mobarhan S (1978) Early detection of cancer of oesophagus along the Caspian Littoral. Report of a pilot project. *Lancet* **1**: 125–126

Dowlatshahi K and Miller RJ (1985) Role of opium in esophageal cancer: a hypothesis. *Cancer Res* **45**: 1906–1907

Farhud DD, Aramesh B, Sawhney KS, Amirshahi P and Daneshmand P (1979) Hp, Tf, Cp and C3 phenotypes in patients with oesophageal cancer and the control group. *Iranian J Public Health* **8**: 111–116

Ghadirian P, Stein GF, Gorodetzky C, Roberfroid MB, Mahon GAT, Bartsch H and Day NE (1985) Oesophageal cancer studies in the Caspian Littoral of Iran: some residual results including opium use as a risk factor. *Int J Cancer* **35**: 593–597

Habibi A (1965) Cancer in Iran. A survey of the most common cases. *J Natl Cancer Inst* **34**: 553–569

Haghighi P, Nabizadeh I, Asvadi SH and Mohallatee EA (1971) Cancer in Southern Iran. *Cancer* **27**: 965–977

Hashemi S, Dowlatshahi K, Day NE, Kmet J, Takasugi M, Mohagheghpour N and Modabber FZ (1979) Esophageal cancer studies in the Caspian Littoral of Iran: introductive assessment of the HLA profile in patients and controls. *Tissue Antigens* **14**: 422–425

Hewer T, Rose E, Ghadirian P, Castegnaro M, Bartsch M, Malaveille C and Day N (1978) Ingested mutagens from opium and tobacco pyrolysis products and cancer of the esophagus. *Lancet* **2**: 494–496

Hormozdiari H, Day NE, Aramesh B and Mahboubi E (1975) Dietary factors and esophageal cancer in the Caspian Littoral of Iran. *Cancer Res* **35** (11 Pt. 2): 3493–3498

IARC (1975) *Annual Report*, pp 115–121. International Agency for Research on Cancer: Lyon

Joint Iran–International Agency for Research on Cancer Study Group (1977) Esophageal cancer studies in the Caspian Littoral of Iran: results of population studies – a prodrome. *J Natl Cancer Inst* **59**: 1127–138

Kirk RL, Keats B, Blake NM, McDermid M, Ala F, Karimi M, Nickbin B, Shabazi H and Kmet J (1976) Genes and people in the Caspian Littoral: a population genetic study in northern Iran. *Am J Phys Anthropol* **46**: 377–390

Kmet J and Mahboubi E (1972) Esophageal cancer in the Caspian Littoral of Iran: initial studies. *Science* **175**: 846–853

Kmet J, McLaren DS and Siassi F (1981) Epidemiology of esophageal cancer with special reference to nutritional studies among the Turkoman of Iran. *Advances in Modern Human Nutrition*, pp 343–365. Pathotex: New York

Mahboubi EO and Aramesh B (1980) Epidemiology of esophageal cancer in Iran, with special reference to nutritional and cultural aspects. *Prev. Med.* **9**: 613–621

Mahboubi E, Kmet J, Cook PJ, Day NE, Ghadirian P and Salmasizadeh S (1973) Oesophageal cancer studies in the Caspian Littoral of Iran: The Caspian Cancer Registry. *Br J Cancer* **28**: 192–214

Munoz N and Day NE (1996) Esophageal cancer. In *Cancer Epidemiology and Prevention*, Schottenfeld D and Fraumeni JF Jr (eds) pp) 681–706. Oxford University Press: New York

O'Neill CH, Hodges GM, Riddle PN, Jordan PW, Newman RH, Flood RJ and Toulson EC (1980) A fine fibrous silica contaminant of flour in the high oesophageal cancer area of north-east Iran. *Int J Cancer* **15**: 617–628

Pour P and Ghadirian P (1974) Familial cancer of the esophagus in Iran. *Cancer* **33**: 1649–1652

Roth MJ, Shu-Fan L, Dawsey SM, Zhou B, Copeland C, Wang GQ, Solomon D, Baker SG, Giffen CA and Taylor PR (1997) Cytologic detection of esophageal squamous cell carcinoma and precursor lesions using balloon and sponge samplers in asymptomatic adults in Linxian, China. *Cancer* **80**: 2047–2059

Sadeghi A, Bhemard Sh, Shafiepoor H and Zeighmani E (1977) Cancer of the esophagus in southern Iran. *Cancer* **40**: 841–845

 *© 2000 Cancer Research Campaign*
